# The Current Waist Circumference Cut Point Used for the Diagnosis of Metabolic Syndrome in Sub-Saharan African Women Is Not Appropriate

**DOI:** 10.1371/journal.pone.0048883

**Published:** 2012-11-08

**Authors:** Nigel J. Crowther, Shane A. Norris

**Affiliations:** 1 Department of Chemical Pathology, National Health Laboratory Service, University of the Witwatersrand Faculty of Health Sciences, Johannesburg, South Africa; 2 MRC/University of the Witwatersrand Developmental Pathways for Health Research Unit, Department of Paediatrics, University of the Witwatersrand Faculty of Health Sciences, Johannesburg, South Africa; John Hopkins bloomerg school of public health, United States of America

## Abstract

The waist circumference cut point for diagnosing the metabolic syndrome in sub-Saharan African subjects is based on that obtained from studies in European populations. The aim of this study was to measure the prevalence of obesity and related metabolic disorders in an urban population of African females, a group at high risk for such diseases, and to determine the appropriate waist cut point for diagnosing the metabolic syndrome. Anthropometry and fasting lipid, glucose and insulin levels were measured in a cohort of 1251 African females participating in the Birth to Twenty cohort study in Soweto, Johannesburg. The waist circumference cut points for diagnosing metabolic syndrome (as defined using the new harmonised guidelines), insulin resistance, dysglycaemia, hypertension and dyslipidaemia were obtained using receiver operator characteristic curve analysis. The prevalence of obesity, type 2 diabetes and metabolic syndrome were 50.1%, 14.3% and 42.1%, respectively. The appropriate waist cut point for diagnosing metabolic syndrome was found to be 91.5 cm and was similar to the cuts points obtained for detecting increased risk of insulin resistance (89.0 cm), dysglycaemia (88.4 cm), hypertension (90.1 cm), hypo-high density lipoproteinaemia (87.6 cm) and hyper-low density lipoproteinaemia (90.5 cm). The present data demonstrates that urban, African females have a high prevalence of obesity and related disorders and the waist cut point currently recommended for the diagnosis of the metabolic syndrome (80.0 cm) in this population should be increased to 91.5 cm. This latter finding demonstrates a clear ethnic difference in the relationship between abdominal adiposity and metabolic disease risk. The similar waist cut points identified for the detection of the individual components of the metabolic syndrome and related cardiovascular risk factors demonstrates that the risk for different metabolic diseases increases at the same level of abdominal adiposity suggesting a common aetiological pathway.

## Introduction

Obesity and its associated diseases are becoming a major public health problem across Africa and in other low- or middle-income countries [1]. Epidemiological studies have shown a distinct gender difference in the prevalence of obesity in African nations, with levels much higher in females than males [2], [3]. This is in contrast to data from high-income countries, where the prevalence of obesity is similar across genders [4]. Data from African studies also show that obesity is most prevalent in urban, middle-aged females [2], [3].

The metabolic syndrome is a grouping together of cardiovascular disease (CVD) risk factors that includes as one of its diagnostic criteria, an elevated waist circumference [5]. The International Diabetes Federation (IDF) guidelines [6] and the new harmonized guidelines for the diagnosis of metabolic syndrome [5] include population specific cut points for waist circumference. However, values for waist circumference in African populations have not been defined due to lack of appropriate data, and therefore, it has been recommended that the cut points derived from European population groups are used for African subjects [5], [6].

The initial values produced for waist circumference cut points were derived in European subjects by calculating the waist circumference levels (≥94 and ≥102 cm in men; ≥80 and ≥88 cm in women) that best predicted a BMI ≥25 and a BMI ≥30, respectively [7]. The lower cut point levels (≥94 for men and ≥80 for women) were incorporated as diagnostic criteria for the metabolic syndrome by the European Group for the Study of Insulin Resistance (EGIR) [8] whilst the National Cholesterol Education Program (NCEP) used the higher cut points in their definition of the metabolic syndrome [9]. The IDF used the same waist cut points as the EGIR for diagnosing the metabolic syndrome in European subjects but used lower waist levels in Asian males [6]. This decision was taken because studies had demonstrated that disease risk in Asian subjects was high at waist circumference cut points below those defined in European populations [10], [11]. However, very little data is available from African populations to show that the current values used for diagnosing excessive abdominal fat deposition, as taken from European cohorts, are appropriate for defining metabolic syndrome in African subjects [12], [13].

The purpose of the present study was to measure the prevalence of metabolic syndrome and each of its individual components in a cohort of middle-aged African, urban females. This population group was chosen because of its high reported prevalence of obesity [2], [3] and hence an assumed high risk of metabolic disease. Additionally, we attempted to define a more suitable waist circumference cut point for the diagnosis of the metabolic syndrome within this population by using receiver operating characteristic (ROC) curve analysis and logistic regression. We also analysed the relationship between waist circumference and the risk of each individual component of the metabolic syndrome and related CVD risk factors. These investigations are important because it is possible that the current waist circumference cut-points for diagnosing metabolic syndrome in sub-Saharan African populations are leading to falsely elevated prevalence rates [13] and little data is currently available on the relationship between waist circumference and metabolic disease risk within this population group [12].

## Materials and Methods

### Participants

The Birth to Twenty (Bt20) cohort started in 1989 with pilot studies to test the feasibility of a long-term follow-up of mothers and their children for the study of their health and wellbeing [14]. Women were enrolled in their second and third trimester of pregnancy through public health facilities and interviewed regarding their health and social history and current circumstances. Singleton children (*n = *3,273) born between April and June 1990 and resident for at least 6 months in the municipal area of Soweto-Johannesburg were enrolled into the birth cohort and have now been followed up 16 times between birth and 20 years of age [15]. Attrition over two decades has been comparatively low (30%), mostly occurring during children’s infancy and early childhood, and approximately 2,300 children and their families remain in contact with the study [16]. The cohort is representative of the demographic parameters of urban South Africa.

During an annual data collection wave, biological mothers participating in the Bt20 cohort were invited to visit a data collection facility either at Chris Hani Baragwanath Hospital in Soweto or the University of the Witwatersrand Medical School in Johannesburg and of those invited 2,174 presented at the facility. Of the mothers who participated, 1,251 were eligible for inclusion in this study as they fulfilled the criteria of being African women and resident in the urban township of Soweto. All data collection has received clearance by the Ethics Committee on Human Subjects at the University of the Witwatersrand (M010556).

### Anthropometric and Blood Pressure Measurements

Body weight and height, in light dress and without shoes, was measured using an electronic scale and a fixed wall stadiometer (Holtain, UK). The waist circumference was measured with a soft measuring tape to the nearest 0.5 cm at the level of the smallest girth above the umbilicus in the standing position. The hip circumference was measured over the widest part of the gluteal region and the waist-to-hip ratio (WHR), was calculated. Blood pressure was measured three times using a digital device (Omron M6; Omron, Kyoto, Japan). Appropriate cuff sizes were used, and participants were measured seated and resting with a several minute break between measurements. The first blood pressure measurement was discarded and the second and third averaged.

### Biochemical Measurements and Disease Diagnosis

Fasting blood samples were obtained from an antecubital vein with minimum stasis. Plasma and serum samples were obtained by centrifugation at 200×g for 20 minutes at 4°C and aliquots were stored at −70°C until assayed.

Total cholesterol, HDL, LDL triglyceride, and glucose concentrations were measured on an auto-analyser (Randox Daytona Clinical Analyzer Randox Laboratories, UK) using enzymatic methods. Insulin (cross-reactivity with proinsulin 8.5%) was assayed on an Immulite analyzer (Siemens Chemiluminescent Technology), and insulin resistance was calculated from fasting glucose and insulin levels, using the homeostasis model assessment (HOMA) method [17].

Metabolic syndrome was diagnosed using the new harmonized guidelines of the International Diabetes Federation; National Heart, Lung, and Blood Institute; American Heart Association; World Heart Federation; International Atherosclerosis Society and International Association for the Study of Obesity [5]. Type 2 diabetes was diagnosed by the presence of a fasting glucose ≥7.0 mmol/l and pre-diagnosed diabetes was identified on the basis of the current use of any physician-prescribed anti-diabetic therapy.

### Statistical Analyses

Data that was not normally distributed was transformed to normality using log or reciprocal values and is presented as a median value with an interquartile range. Data with a normal distribution is presented as mean ± SD.

Optimal waist cut-off values for diagnosing metabolic syndrome, and for each of the biochemical and blood pressure components of the metabolic syndrome [5], were determined using receiver operator characteristic (ROC) curve analysis. In this analysis, metabolic syndrome was diagnosed if 3 of the following 4 criteria were present: fasting glucose ≥5.6 mmol/L; fasting triglyceride ≥1.7 mmol/l; fasting HDL <1.3 mmol/l and systolic ≥130 and/or diastolic ≥85 mmHg. Optimal waist cut-off values for identifying subjects with insulin resistance (HOMA levels in the top quartile) raised fasting LDL (≥3.0 mmol/l) and total cholesterol levels (≥5.0 mmol/l) were also determined using ROC curve analysis. The optimal waist cut-off value was defined using the Youden Index [18].

In order to determine whether our sample size was appropriate, we used a null hypothesis of the area-under-the-ROC curve being 0.5 [19]. Assuming a 33% prevalence level for metabolic syndrome and a conjectured area-under-the-ROC curve of 0.6, with α set at 0.05 and β at 0.10 (90% power), the study would require a sample number of approximately 305 subjects. This was well below our actual sample size (see Results).

Logistic regression was used to determine whether the waist cut-off values obtained from the ROC curves occurred at true inflection points. For each of the variables described above subjects were divided into 4–8 roughly equal sized groups based on waist circumference and including one group for which the lower cut-point value was the same as the optimal waist circumference level obtained from the ROC curve analysis. Logistic regression, adjusted for age, was then performed to determine if there was any significant increase in disease risk at the optimal waist cut point. The percentage prevalence of each metabolic disorder was also calculated in each of these waist circumference-defined groups and compared using the χ^2^ test.

## Results

### Characteristics of Study Population

As shown in Table 1, complete data were not available on all 1,251 study participants with blood analyte data obtained from between 474–609 subjects. However, subjects for whom metabolic data were available did not differ in terms of anthropometry from those for whom data were not available.

**Table 1:** Anthropometric and fasting biochemical parameters in study population.

Variables	Level in study population	N
Age (years)	40.0 [10.6]	1,251
BMI	30.5±6.69	1,251
Waist (cm)	87.4±13.3	1,237
Systolic (mmHg)	113 [24.0]	1,180
Diastolic (mmHg)	74.5 [16.0]	1,180
Triglyceride (mmol/l)	0.92 [0.83]	609
Total cholesterol (mmol/l)	3.50±1.69	609
LDL (mmol/l)	1.33 [0.83]	607
HDL (mmol/l)	0.97 [0.70]	608
Insulin (pmol/l)	6.84 [5.49]	474
Glucose (mmol/l)	5.10 [1.28]	746
HOMA	1.52 [1.32]	449

Data expressed as mean ± SD or median [interquartile range].

The principle characteristic of this study cohort was their high mean BMI of 30.5±6.69, with an obesity prevalence of 50.1% (see Table 2). Abdominal obesity was also very common with 69.3% of the population having a waist circumference ≥80 cm. The prevalences of metabolic syndrome (42.1%), diabetes (14.3%) and fasting HDL levels below 1.3 mmol/l (70.1%), were also alarmingly high.

**Table 2:** Prevalence of type 2 diabetes, metabolic syndrome and related metabolic abnormalities in study population.

Disorder	Prevalence
Type 2 diabetes	14.3%
Impaired fasting glucose	20.3%
Metabolic syndrome	42.1%
Systolic ≥130 and/or diastolic ≥85 mmHg	36.4%
Triglyceride ≥1.7 mmol/l	22.3%
HDL <1.3 mmol/l	70.1%
Total cholesterol ≥5.0 mmol/l	15.4%
LDL ≥3.0 mmol/l	2.8%
Waist ≥80 cm	69.3%
BMI ≥30	50.1%

### Identification of Waist Circumference Cut Points for Diagnosing Metabolic Syndrome and Related Disorders

Receiver operating characteristic (ROC) curve analysis was used to determine the optimal waist circumference cut points for diagnosing metabolic syndrome and its related disorders. Table 3 gives the area under the ROC curve for each of these disorders with waist circumference acting as the diagnostic variable. These data show that the presence of metabolic syndrome, insulin resistance, LDL ≥3.0 mmol/l, hypertension, HDL <1.3 mmol/l and dysglycaemia can all be identified to varying degrees of accuracy using waist circumference. However, this is not the case for cholesterol ≥5.0 mmol/l or triglycerides ≥1.7 mmol/l. Table 3 also gives the optimal waist circumference cut points that were identified from ROC curve analysis for diagnosing each metabolic disorder, along with the sensitivity and specificity of each of these cut points. It is interesting to note that with the exception of hypertriglyceridaemia, all the optimal waist cut points identified for diagnosing each of the metabolic syndrome-related disorders, fall within a fairly narrow range (87.6–91.5 cm).

**Table 3:** Characteristics of ROC curves used for the identification of optimum waist circumference cut-off values for the diagnosis of metabolic syndrome, insulin resistance, hypertension, dysglycaemia and dyslipidaemia.

Test variable	Area under ROC curve	Optimal waist cut-off value	Sensitivity	Specificity
Presence of metabolic syndrome	0.74 (0.67–0.81)**	91.5 cm	0.69 (0.55–0.81)	0.72 (0.68–0.75)
HOMA level in top quartile	0.68 (0.61–0.74)**	89.0 cm	0.62 (0.51–0.72)	0.65 (0.59–0.71)
LDL ≥3.0 mmol/l	0.66 (0.55–0.77)*	90.5 cm	0.65 (0.38–0.86)	0.64 (0.60–0.68)
Systolic ≥130 and/or diastolic ≥85 mmHg	0.63 (0.60–0.67)**	90.1 cm	0.52 (0.47–0.57)	0.69 (0.66–0.72)
HDL <1.3 mmol/l	0.61 (0.56–0.65)**	87.6 cm	0.53 (0.48–0.58)	0.67 (0.60–0.73)
Glucose ≥5.6 mmol/l	0.59 (0.55–0.63)**	88.4 cm	0.56 (0.50–0.61)	0.59 (0.54–0.63)
Cholesterol ≥5.0 mmol/l	0.54 (0.48–0.60)	90.5 cm	0.44 (0.34–0.54)	0.64 (0.60–0.68)
Triglyceride ≥1.7 mmol/l	0.48 (0.42–0.53)	79.0 cm	0.72 (0.64–0.79)	0.30 (0.26–0.34)

Figures in parentheses are 95% confidence intervals; *P<0.005, **P<0.0001 for hypothesis test of whether area under the ROC curve is greater than 0.5.

The ROC curve analysis demonstrates that the optimal waist cut point for diagnosing metabolic syndrome in this population group is 91.5 cm (see Table 3). If this cut-point is incorporated into the criteria put forward under the new harmonised guidelines [5], then the prevalence of metabolic syndrome is 26.2%, as compared to 42.1% observed using the waist cut-point of 80 cm. If one uses the current IDF guidelines for diagnosing metabolic syndrome [6] then the prevalence is 40.1%.

### The Relationship between Waist Circumference and the Risk of Metabolic Disease

The waist circumference cut points identified from ROC curve analyses were further investigated to determine whether they occurred at true inflection points for disease prevalence and risk. The data in Table 4 demonstrates that at a waist circumference cut point of 91.5 cm, both the prevalence and risk of metabolic syndrome increase significantly. Similar trends were observed for insulin resistance at a waist circumference cut point of 89 cm (Table 5), hypertension at 90.1 cm (Table 6), HDL levels <1.3 mmol/l at 87.6 cm (Table 7) and dysglycaemia at 88.4 cm (Table 8). The waist cut point for identifying subjects with LDL levels ≥3.0 mmol/l could not be analysed in a similar fashion due to the very low prevalence (2.8%).

**Table 4:** Relationship between waist circumference and percentage of subjects with and risk of, metabolic syndrome (MetS).

Group	Waist circumference (cm)	N	Percentage with MetS	Risk of MetS
1	<73.0	124	2.4*** ††	1.0
2	73.0–79.9	116	1.7*** ††	0.64 (0.1–4.0)
3	80.0–85.9	120	5.8**	2.2 (0.5–8.8)
4	86.0–91.4	107	4.7**	1.5 (0.3–6.6)
5	91.5–98.9	102	11.8*	3.9 (1.0–14.6)‡
6	>98.9	102	23.5	8.4 (2.4–29.6)‡‡

Risk is expressed as an odds ratio (95% confidence intervals) compared against group 1, with adjustment for age;

* P<0.05,

** P<0.005,

*** P<0.0001 versus group 6;

†† P<0.005 versus group 5;

‡ P<0.05,

‡‡ P<0.005 versus group 1.

**Table 5:** Relationship between waist circumference and percentage of subjects with and risk of, insulin resistance.

Group	Waist circumference (cm)	N	Percentage with insulin resistance	Risk of insulin resistance
1	<74.0	66	12.1*** †† ‡	1.0
2	74.0–78.9	59	11.9*** †† ‡	0.9 (0.3–2.8)
3	79.0–83.9	57	22.8*	1.9 (0.7–5.2)
4	84.0–88.9	57	19.3** †	1.6 (0.6–4.4)
5	89.0–94.9	61	31.1	3.3 (1.3–8.5)§
6	95.0–1010	63	34.9	3.5 (1.4–8.8)§
7	>1010	69	45.8	6.3 (2.5–15.9)§§§

Risk is expressed as an odds ratio (95% confidence intervals) compared against group 1, with adjustment for age;

* *P*<0.05,

** *P*<0.005,

*** *P*<0.0001 versus group 7;

† *P*<0.05,

†† *P*<0.005 versus group 6;

‡ *P*<0.05 versus group 5;

§ *P*<0.05,

§§§ *P*<0.0001 versus group 1.

**Table 6:** Relationship between waist circumference and percentage of subjects with and risk of, hypertension.

Group	Waist circumference (cm)	N	Percentage with hypertension	Risk of hypertension
1	<72.0	144	20.8*** †† ‡‡	1.0
2	72.0–76.9	165	20.6*** †† ‡‡‡	1.0 (0.5–1.7)
3	77.0–82.4	141	27.7*** † ‡	1.3 (0.7–2.3)
4	82.5–85.9	148	31.1*** ‡	1.5 (0.8–2.5)
5	86.0–90.0	150	29.3*** ‡	1.2 (0.7–2.2)
6	90.1–95.9	137	43.1**	2.1 (1.3–3.6)§§
7	96.0–101.5	158	39.2**	2.0 (1.1–3.4)§
8	>101.5	148	60.1	4.1 (2.4–7.1)§§§

Risk is expressed as an odds ratio (95% confidence intervals) compared against group 1, with adjustment for age;

** P<0.005,

*** P<0.0001 versus group 8;

† P<0.05,

†† P<0.005 versus group 7;

‡ P<0.05,

‡‡ P<0.005,

‡‡‡ P<0.0001 versus group 6;

§ P<0.05,

§§ P<0.005,

§§§ P<0.0001 versus group 1.

**Table 7:** Relationship between waist circumference and percentage of subjects with and risk of, hypo-high density lipoproteinaemia.

Group	Waist circumference (cm)	N	Percentage with hypo-HDL	Risk of hypo-HDL
1	<74.0	107	62.6** †	1.0
2	74.0–81.9	99	62.6** †	1.0 (0.6–1.7)
3	82.0–87.5	102	60.8** †	0.9 (0.5–1.6)
4	87.6–97.0	135	74.8	1.7 (1.0–3.0)§
5	>97.0	137	82.5	2.6 (1.4–4.8)§§

Risk is expressed as an odds ratio (95% confidence intervals) compared against group 1, with adjustment for age;

** P<0.005 versus group 5;

† P<0.05 versus group 4;

§ P = 0.06,

§§ P<0.005 versus group 1.

**Table 8:** Relationship between waist circumference and percentage of subjects with and risk of, dysglycaemia.

Group	Waist circumference (cm)	N	Percentage with dysglycaemia	Risk of dysglycaemia
1	<75.0	131	23.7*** † ‡	1.0
2	75.0–82.4	124	29.0**	1.4 (0.8–2.4)
3	82.5–88.3	127	29.9**	1.4 (0.8–2.4)
4	88.4–94.4	117	38.5	2.0 (1.1–3.5)§
5	94.5–1010	120	37.5	1.9 (1.1–3.3)§
6	>1010	109	49.5	3.1 (1.7–5.6)§§

Risk is expressed as an odds ratio (95% confidence intervals) compared against group 1, with adjustment for age;

** P<0.005,

*** P<0.0001 versus group 6;

† P<0.05 versus group 5;

‡ P<0.05 versus group 4;

§ P<0.05,

§§ P<0.005 versus group 1.

## Discussion

South Africa is a middle income country, as defined by the World Bank [20], and it shares with other developing African nations key epidemiological features of the nutritional transition. Thus, the prevalence of obesity is high, particularly in urban areas [2], [21] and is more prevalent in females than males [2], [3]. Therefore, the current investigation was performed in an urban population of middle-aged, African females residing in the Soweto-Johannesburg conurbation, a group that would be presumed to have a high prevalence of obesity and co-morbid diseases. Our results clearly confirm that this population does have a high prevalence of obesity, metabolic syndrome and related disorders and further emphasises the high burden of disease imparted by obesity-related disorders within low- or middle-income countries [1].

The high prevalence of metabolic syndrome within this study cohort is largely driven by the high levels of abdominal obesity and the low fasting HDL serum concentrations, as reported in other epidemiological surveys of metabolic syndrome in African populations [19], [22]. The very low prevalence of high LDL-cholesterol serum levels in this study population (2.8%) is not unexpected. It has previously been shown that LDL levels are lower in African compared to European and Indian populations within South Africa [23].

The prevalence of obesity in this population is 50%, which is the highest yet recorded in an indigenous, sub-Saharan African population. Obesity is known to be increasing in the developing world as ‘westernization’ of dietary intakes and urbanisation of former rural populations lead to significant changes in lifestyle [24], [25]. Thus, studies in South Africa have shown that dietary fat intake has increased significantly over the preceding 5 decades, and that urbanisation is associated with a higher prevalence of obesity [24], [26]. The genetic aetiology of obesity in African populations is currently uncertain, with most genome-wide association studies having been conducted in European cohorts. It is therefore not possible for us to rule out a genetic component to the high BMIs observed within our study population. Type 2 diabetes and impaired fasting glucose were also found to exist at high levels within this population group. This may largely be due to the high prevalence of obesity, particularly abdominal obesity, and the age of the study cohort.

The high frequency at which obesity and co-morbid diseases occur in this study population further confirms the growing problem of diseases of lifestyle in low and middle-income countries [1]. Across sub-Saharan Africa the prevalence of diabetes [27], hypertension [28] and coronary artery disease [29] are known to be increasing and this is mirrored by rising levels of obesity [3], [21]. The financial and social burden of non-communicable diseases within resource limited nations is leading to the further deterioration of over-stretched public health services that are already compromised by an epidemic of communicable diseases [30]. Intervention at the primary health care level is therefore essential. However, because obesity is not readily acknowledged as a health problem in some African populations [31], [32], lifestyle modification methods, as developed in higher income countries, may not be applicable in this environment without extensive education programs.

The IDF [6] and the new harmonised guidelines [5] for the diagnosis of the metabolic syndrome both include waist cut-off points for different ethnic groups and recommend that for sub-Saharan African populations the European waist cut-off points are used. However, it is known that for a given waist circumference African females have less visceral fat than European females and it has therefore been suggested that if waist threshold levels are defined by visceral fat mass then they should be different for these 2 population groups [33]. Only one other large epidemiological study has been undertaken to determine the optimal waist cut-off points for sub-Saharan African subjects [13]. This study was also performed in South Africa, in a rural cohort of 947 male and female subjects and demonstrated that for diagnosis of the metabolic syndrome, a waist circumference cut point of 92 cm for women, was optimal. This figure, obtained using ROC curve analysis, is very similar to that reported in the present study (91.5 cm). However, it is important to note that there are considerable differences between the populations used in these two studies. The present investigation used an urban population of female subjects with a minimum age of 28 years and an obesity prevalence of 50.1%, whereas the study of Motala *et al.* [13] analysed female subjects from a rural environment with a minimum age of 16 years and an obesity prevalence of 22.6%. Despite these differences, almost the same waist cut points were obtained for diagnosing the metabolic syndrome. This suggests that, irrespective of differences in lifestyle and obesity prevalence levels, the same cut point for waist circumference can be applied across these population groups. It should also be noted that in a smaller study of urban African male (n = 80) and female (n = 93) teachers, ROC curve analysis showed that the optimal waist cut point for the diagnosis of the metabolic syndrome in females was 98 cm [34].

A very important conclusion of this study, which is supported by data from the two investigations discussed above, is that the waist circumference cut-point currently being utilised for the diagnosis of metabolic syndrome in sub-Saharan African females (80 cm) [5], [6] is too low and will therefore give an over estimation of prevalence. This is demonstrated in the current study where, using the waist cut point of 91.5 cm incorporated into the new harmonised guidelines [5], the prevalence of metabolic syndrome is 26.2% compared to 42.1% when using the cut point of 80 cm. If one uses the current IDF criteria for diagnosis, then the prevalence of metabolic syndrome is 40.1%. This hypothetical fall in the prevalence of metabolic syndrome may have important implications for any national intervention programs aimed at subjects with an elevated waist circumference, as it would lead to a reduction in the number of subjects requiring therapy. This would be important in low and middle income countries such as South Africa, where financial resources and infrastructure are constrained. It would also ensure that therapies are targeted to those at greatest risk of developing CVD or diabetes.

The finding that the waist cut-point currently recommended for the diagnosis of the metabolic syndrome is not applicable to sub-Saharan African populations highlights the inappropriate use of guidelines derived from non-African study cohorts for the diagnosis of diseases within Africa. This is further exemplified by a study showing that an HbA1c cut point of 6.5% for the diagnosis of diabetes, as derived from studies in which no sub-Saharan populations were included [35], was not applicable within a mixed ancestry group from South Africa [36].

The rising prevalence of obesity [3], [21] and co-morbid diseases [27]–[29] within sub-Saharan Africa suggests that an inevitable rise in the prevalence of the metabolic syndrome must also be occurring. Furthermore, the HIV epidemic within these nations may also be contributing to the high prevalence of metabolic diseases since African-based studies have shown that HIV and anti-retroviral therapy are both linked to metabolic dysfunction [37]–[39]. Given these circumstances, the determination of the appropriate waist cut-point for diagnosing metabolic syndrome in sub-Saharan populations becomes vitally important as it will allow for a more accurate estimation of the changing prevalence levels of the syndrome over time and in response to interventions.

An interesting and important feature of our study is that the prevalence and the risk of a number of different metabolic disorders increase at a very similar waist circumference cut-point. This may be related to visceral adiposity because in previous studies this body fat depot has been linked to all the metabolic variables analysed in the current investigation [40]. Furthermore, it has been suggested that there is a level of visceral fat above which there is increased cardiovascular risk [41], and it is therefore possible that in African females a waist circumference of 87.6–91.5 cm equates to this visceral fat level. There is an alternative hypothesis which suggests that the insulin sensitivity of the subcutaneous fat depot determines the threshold of triglyceride deposition within that tissue [42]. Once this threshold is exceeded (equivalent to a waist circumference of 87.6–91.5 cm?), triglycerides will be deposited at other sites including the visceral adipose depot, skeletal muscle and liver, and it is this ectopic fat deposition that leads to an increased risk of metabolic disease.

Waist circumference has a relatively poor sensitivity and specificity for diagnosing metabolic disorders, as shown in this and other investigations [43], [44]. Waist circumference is a proxy indictor of visceral fat mass [45] and it has been shown that the latter anthropometric variable is a better diagnostic criterion for identifying metabolic syndrome than the former [46]. However, the measurement of waist circumference is far simpler and less expensive than that of visceral fat and is therefore the preferred anthropometric component in all diagnostic guidelines for metabolic syndrome.

There are some limitations to this study. Lipid, glucose and insulin levels were not obtained in all subjects, however there were no differences in anthropometric variables between subjects who did or did not have these analytes measured. Despite metabolic data not being obtained for all subjects, the study was still sufficiently powered to demonstrate that the area under the ROC curve for metabolic syndrome, insulin resistance, hypertension, hypo-high density lipoproteinaemia and dysglycaemia were statistically significantly greater than 0.5. It has been suggested that the ability to statistically demonstrate that the area under the ROC curve is greater than 0.5 is the most appropriate way to determine the correct sample size for a ROC curve analysis [19]. Another limitation of the study is that the population investigated was a stable, urban group and therefore may not be comparable to rural populations. However, as previously noted, a study performed in a rural South African population did obtain a very similar optimal waist cut point for the diagnosis of metabolic syndrome to that obtained in the current study [13]. Our study only included females and therefore further investigations must be performed in male subjects.

In conclusion, urban, middle-aged African females have very high prevalence levels of abdominal obesity, type 2 diabetes and metabolic syndrome. The reason for the high prevalence of these diseases is not known however, lifestyle factors may play a major role but a genetic aetiology cannot be ruled out. The determination, by ROC curve analysis, of optimal waist circumference cut points for the diagnosis of the metabolic syndrome demonstrated that a value of 91.5 cm would be more appropriate than the currently recommended level of 80 cm. Further investigations are necessary to determine whether this cut point can also be used in other sub-Saharan African populations.
